# A New Approach to Studying the Mechanical Characteristics of the Anchoring–Grouting System in Broken Surrounding Rock

**DOI:** 10.3390/s23218931

**Published:** 2023-11-02

**Authors:** Lei Wang, Wei Lu

**Affiliations:** 1 College of Civil Engineering, Ludong University, Yantai 264025, China; mbflove@ldu.edu.cn; 2 Research Center of Geotechnical and Structural Engineering, Shandong University, Jinan 250061, China

**Keywords:** fractured surrounding, anchoring–grouting system, bearing capacity, mechanical characteristics, cooperative bearing

## Abstract

With the increasing depletion of shallow coal resources, deep roadway excavation has become the main direction in the development of coal mining. Due to geological conditions including high stress and extremely broken rock, disasters such as squeezing, bulging, and swelling are widely observed. The anchoring–grouting support method is one of the most effective methods of surrounding rock reinforcement. To study the mechanical characteristics of the anchoring–grouting system in broken surrounding rock, laboratory tests considering the water–cement ratio and preload were carried out. The research results show that the internal force of support and the deformation of the support surface have close relationships with the bearing stages of the anchoring–grouting system. The optimal water–cement ratio and a higher preload can improve the cooperative bearing characteristics of surrounding rock and its support, which is of great significance for enhancing the strength of surrounding rock and reducing roadway deformation. The research results can provide a reference for anchoring–grouting support design in deep roadway excavation.

## 1. Introduction

**Figure 1:**
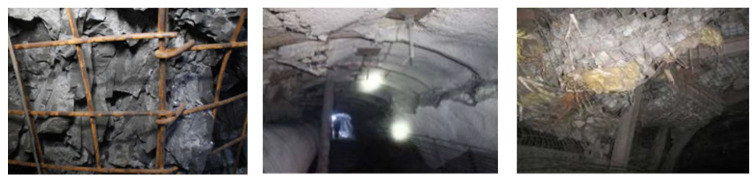
Failure phenomenon of broken surrounding rock in deep coal mine roadway.

The coal industry accounts for 59% of the primary energy in China. As the “Belt and Road” is developing rapidly worldwide, coal consumption will continue to grow in the future. However, with the increasing depletion of shallow coal resources, deep roadway excavation has become the main direction in the development of coal mining [1]. In the deep strata, after the roadway is excavated, affected by the overlying loads, the integrity of the surrounding rock is destroyed, and the self-supporting capacity is lost. Due to geological characteristics such as high stress and extremely broken rock, the surrounding rock of the mining roadway exhibits low strength at the engineering scale (Figure 1). Disasters including squeezing, bulging, and swelling in deep roadways are widely observed, which seriously affect personnel safety and economic benefits in deep roadway construction, restrict the rapid tunneling of roadways, and threaten the safe production of coal mines [2,3,4].

The anchorage capacity of fully grouted bolts has been studied for many years. However, the improvement of the strength of fractured rock masses after anchoring–grouting reinforcement cannot be quantified yet, and therefore there is still a lack of scientific methodology in understanding the collaborative interaction between the anchoring–grouting system and the anchor, as well as the crack characteristics of the anchoring–grouting system [5,6,7,8]. The bearing capacity of the anchoring–grouting system becomes a key factor controlling the stability of surrounding rock [9,10]. Liu aimed at addressing the shortcomings of the normal grouting process and adopted the three-step grouting process for surrounding rock reinforcement. A grouting diffusion depth of surrounding rock of more than 3 m will significantly improve the effect of grouting reinforcement, enhancing the self-bearing capacity of the rock mass and effectively controlling the overall stability [11]. Yang analyzed the influence of the number of anchor bolts and prestress on the fracture characteristics of sandstone and analyzed the crack initiation, propagation, and relationship during the deformation of grouting-anchored fractured sandstone [12]. Zhang conducted experimental research on fractured marble samples after reinforcement by “cement grouting” and “bolt+ grouting” combined with laser scanning and electron microscope scanning, and analyzed the reinforcement effect and action mechanism of the grouting and bolt on the fractured surface of the marble [8]. Su carried out the anchor-bearing test of a rock mass in the fault fracture zone and analyzed the influence of the anchor on the mechanical properties of the damaged rock mass [13]. Wang adopted 3D printing technology to study the slurry diffusion and bubble formation process of grouting pressure [14]. Wang made a visual test system for microcrack grouting, used three kinds of cement slurry materials, studied the slurry filtration efficiency of different cement particle sizes under various crack openings, and established the basic seepage equation of deep–shallow coupling anchor grouting fluid with the help of seepage mechanics theory [15]. Based on deep and shallow grouting reinforcement, and considering the diffusion relationship of the grout, an integrated support technology with bolt grouting support as the core and other supports coordinated with each other is adopted to ensure the stability of the deep roadway [16]. 

Previous research has focused on the anchoring–grouting system without the use of anchoring grouting bolts. Studies on the anchoring–grouting system with anchoring grouting bolts remain limited. Specifically, there is a lack of comprehensive investigation on quantitatively enhancing the bearing capacity of fractured rock masses through reinforcement measures. Given this, this paper takes a typical example of deep mining engineering, the Suncun coal mine in the Xinwen mining area of China, as the engineering background, selects the on-site broken weak rock mass as research samples, processes the anchoring–grouting system specimens, and analyzes the bearing mechanical characteristics of the anchoring–grouting system considering different water–cement ratios and different values of preload. This paper focuses on the displacement relationship of the support face of the reinforced surrounding rock (anchoring–grouting system) and the overall failure mechanism. The interaction relationship between the displacement of the support surface and the properties of the supporting members is clarified.

## 2. Materials and Test Methods

### 2.1. Anchoring–Grouting System Test Scheme

After a deep roadway is excavated, the surrounding rock of the roadway changes from a three-dimensional stress state to a two-dimensional stress state. The surrounding rock stress is adjusted for the first time. After mining the working face, the roadway is affected by dynamic pressure, and the deep roadway is rebalanced for a second time. As a result, the roadway roof becomes loose and broken, its bearing capacity is significantly weakened, the roadway roof keeps sinking, and bolt (cable) failure occurs frequently.

**Figure 2:**
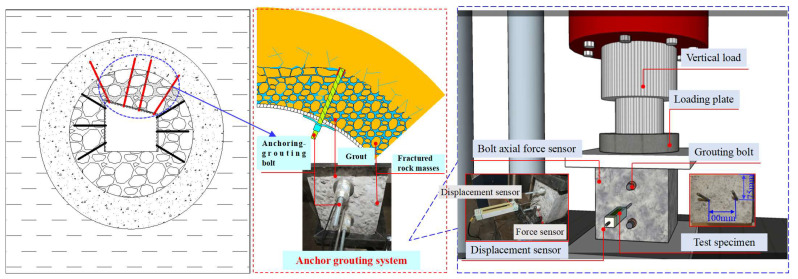
Bearing structure model and test scheme of anchoring–grouting system.

In the Suncun coal mine, the deep roadway is supported by an anchoring grouting bolt, while the grouting slurry cements the fractured surrounding rock into a whole to form a regenerated rock mass with higher bearing capacity. Based on the mechanical characteristics of the anchoring–grouting bearing system of the deep roadway, the analytical bearing structure model of the anchoring–grouting system is established, as shown in Figure 2.

On-site rock samples were collected to make the anchoring–grouting system specimen, the size of which was 150 mm × 150 mm × 200 mm. According to the relevant research results of Yang [12], Φ6 × 200 mm stainless rebar was selected to simulate the support members. Ordinary Portland cement with a compressive strength of 32.5 MPa with no additives was adopted as the grouting material. All the anchoring modes in this study had the form of full-length anchoring support. The layout scheme of supporting members was as follows: the anchoring–grouting system had two supporting members with a distance of 100 mm. The supporting member was in the middle and 5 mm away from the short side containing the tray and nuts to apply preload force to the anchoring–grouting system. 

The specimen preparation process was as follows: Firstly, broken rock with different particle sizes was screened out and placed into the mold, and a stainless-steel threaded bolt with the diameter of 6 mm and length of 200 mm was pre-embedded in the mold. Then, the prepared cement slurry was grouted into the broken rock to form the anchoring–grouting system specimen, which was standardly cured for the next 28 days. Secondly, when applying the preload, the nut was rotated on the anchoring bolt and the preload applied to the support surface of the anchoring–grouting system specimen through a tray. 

Considering the influence of the water–cement ratio (0.4, 0.5, and 0.8) and preload (0 kN, 1 kN, and 1.75 kN), as shown in Table 1, a total of 9 research and comparison schemes were designed to study the relationship of bearing capacity and failure characteristics of the anchoring–grouting system under different influencing factors.

### 2.2. Anchoring–Grouting System Test Procedure

**Figure 3:**
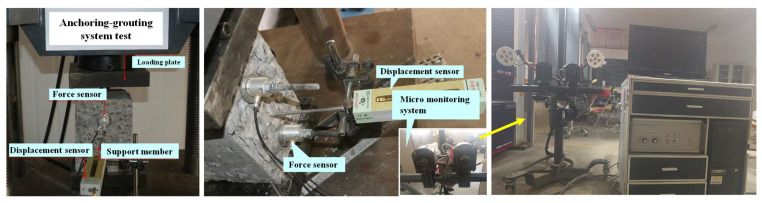
The test scheme of the anchoring–grouting system.

The anchoring–grouting system test equipment comprised the loading system, the displacement monitoring system, the supporting member stress monitoring system, and the monitoring micro-system for cracks. The loading system mainly consisted of two parts: the vertical loading and the preload force loading. The vertical loading of the anchoring system was applied by the electronic precision material machine, with the maximum load of 1000 kN. The preload force was applied to the specimen by rotating the nut. The displacement monitoring system mainly included vertical and horizontal displacement monitoring. The vertical displacement was automatically measured by the electronic precision material machines, and the horizontal displacement was measured by the high-precision displacement sensors in the horizontal direction. The sensor range was 100 mm, with an accuracy of ±0.01 mm, which was installed and fixed in the middle position of the anchoring–grouting system support surface with the magnetic base to achieve real-time monitoring of the deformation of the anchoring–grouting system. The supporting member monitoring system consisted of high-precision pressure micro-sensors and acquisition software, with the pressure sensor range of 10 MPa, achieving real-time monitoring of the anchoring and grouting system’s support component stress. The monitoring microsystem for cracks mainly consisted of a three-dimensional moving observation frame, a DIC microimaging lens, and a three-dimensional large-format light source system, using light source sensors to monitor the characteristics of crack damage in the anchoring–grouting system, as shown in Figure 3.

The test process was as follows: Firstly, we placed the anchoring–grouting system into the electronic precision material machine, adjusted the direction so that the center of the anchoring–grouting system was in a straight line with the pressure head of the testing machine, and then rotated the nut to apply preload force to the anchoring–grouting system. We set the loading rate to 0.01 mm/min, started the testing machine and data collection system, and then started the anchoring–grouting system bearing test.

## 3. Analysis of The Test Results

Relevant studies have tested the bearing characteristics of anchoring–grouting reinforcement under different rock particle sizes, lithologies, and other factors [17,18]. Still, no preload was considered. The preload is an essential embodiment of the active support, which can increase the overall support strength of the roadway. Therefore, it is of great significance to analyze the mechanical characteristics of the anchoring–grouting system with preload applied in order to further reveal the mechanism of the anchoring–grouting system in weak and broken surrounding rock.

To quantitatively compare the cooperative bearing behavior of the anchoring–grouting system under different conditions, the cooperative bearing coefficient of the anchoring–grouting system is defined as *η_i_*:

(1) $$\eta_{i}=\frac{N_{iz}}{N_{ix}}\times 100\%$$

where *N_iz_* is the peak stress of supporting members in each scheme (kN), and *N_ix_* is the peak stress of the anchoring–grouting system (kN).

To quantitatively compare the coordinated deformation of the anchoring–grouting system under different conditions, the coordinated deformation coefficient of the anchoring–grouting system is defined as *ζ_j_*:

(2) $$\zeta_{j}=\frac{D_{jz}}{D_{jx}}\times 100\%$$

where *D_jz_* is the displacement of the support surface corresponding to the peak stress of the supporting members in each scheme (mm), and *D_jx_* is the vertical displacement corresponding to the peak stress of the anchoring–grouting system (mm).

### 3.1. Bearing Mechanical Properties of Anchoring–Grouting System with Different Water–Cement Ratios

#### 3.1.1. Strength Variation Relationship of Anchoring–Grouting System

**Figure 4:**
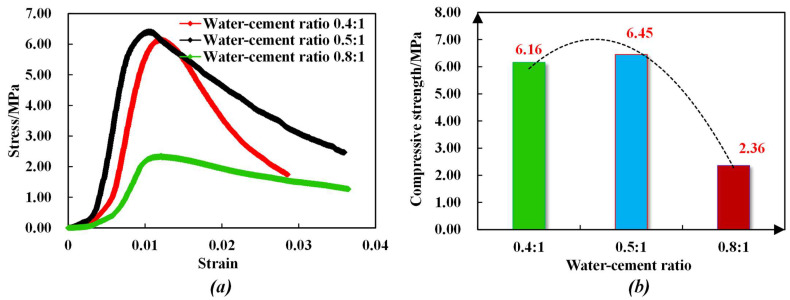
Stress–strain curve of anchoring–grouting system with different water–cement ratios. (**a**) Stress–strain curve. (**b**) Compressive strength histogram.

Figure 4 shows the stress–strain curves and a comparison of the bearing capacities of anchoring–grouting systems with different water–cement ratios. It can be concluded that:

(1) With the increase in the water–cement ratio, the bearing capacity of the anchoring–grouting system first increases and then decreases. When the water–cement ratio changes from 0.4 to 0.5, the compressive strength of the anchoring–grouting system rises by about 7%, and when the water–cement ratio changes from 0.5 to 0.8, the compressive strength of the anchoring–grouting system decreases by about 64%. The relationship between the compressive strength of the anchoring–grouting system and the water–cement ratio presents an obvious quadratic function.

(2) When the water–cement ratio is 0.8, the cement slurry is thinner, and the cement content is less, which directly reduces the bearing capacity of the anchoring–grouting system. When the cement slurry is 0.4, the cement slurry is thicker, the cement content is high, the fluidity is weak, and the filling effect is poor, so the bearing capacity of the anchoring–grouting system is small. A lower water–cement ratio can be used for grouting in the shallow surrounding rock of a deeply broken roadway, while for a deep roadway, with poor fracture development, a higher water–cement percentage can be used.

**Figure 5:**
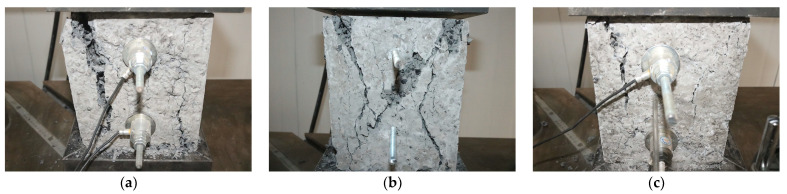
Failure modes of anchoring–grouting system with different water–cement ratios. (**a**) Water–cement ratio: 0.4. (**b**) Water–cement ratio: 0.5. (**c**) Water–cement ratio: 0.8.

Figure 5 shows the failure modes of the anchoring–grouting system with different water–cement ratios obtained by the monitoring microsystem. The crack failure of the anchoring–grouting system is mainly divided into two types: First, tensile force concentrated on the free surface, leading to tensile failure. Second, tensile force predominantly existing in the early stage and then gradually transitioning to a mixed tensile and shear failure, which mainly occurs on the support surface. When the water–cement ratio is 0.5, the specimen shows the phenomenon of tensile–shear failure, and the peak load achieves the highest value, indicating that the strength is maximized in the tension–shear failure type.

#### 3.1.2. Stress Variation Relationship of Supporting Members

**Figure 6:**
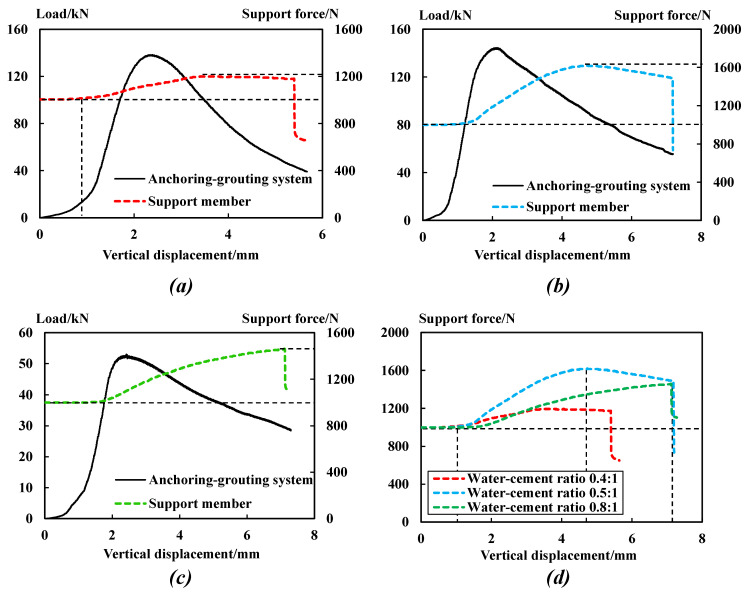
Relationship between the monitored loads/force and the vertical displacement of the anchoring–grouting system and the support members. (**a**) Water–cement ratio: 0.4. (**b**) Water–cement ratio: 0.5. (**c**) Water–cement ratio: 0.8. (**d**) Stress comparison curve of supporting members.

A hollow pressure micro-gauge monitors with high precision the stress changes of the supporting members in real time. Figure 6 shows the relationship between the monitored loads/force and the vertical displacement of the anchoring–grouting system and the support members.

The comparative analysis in Figure 6 shows that:

(1) The stress of the supporting members can be divided into five stages: Steady stage: when the internal force remains the value of the preload. Rising stage: when the internal force starts and then keeps rising due to the increase in deformation trend. Stable or falling stage: when the deformation reaches its limit, due to different levels of support stress, it may continue to stabilize the stress or reach the limit state and decrease. Sudden falling stage: the support members are damaged with no bearing capacity.

(2) From the elastoplastic stage of the anchoring–grouting system, the stress of the supporting members begins to increase. When the load of the anchoring–grouting system reaches the peak, the stress of the supporting members is in the rising stage, while in the post-peak failure stage of the anchoring–grouting system, the stress of the supporting members is still in the increasing and stable stages. The stress of the supporting members remains stable for a certain time, and the supporting members give full play to their support potential. This shows that the supporting members play an essential role after the bearing peak of the anchoring–grouting system.

(3) With the formation of macrocracks after the peak strength of the anchoring–grouting system, the interface bonding effect between the support component and the surrounding rock is damaged, so the stress of the support component gradually reduces. When the support component slips and fails, the stress of the support component drops suddenly.

(4) With the increase in the water–cement ratio, the stress of supporting members increases first and then decreases. The peak stress of the supporting members of specimens with water–cement ratios of 0.4, 0.5, and 0.8 is 1100 N, 1620 N, and 1460 N, respectively, and the stress increase rates of the supporting members are 22%, 62%, and 46%, respectively.

The above phenomenon indicates that when the water–cement ratio is low, the cement slurry is thicker and its filling and diffusion effect in the broken surrounding rock is poor [17], resulting in insufficient contact between the supporting members and the surrounding rock, reducing the interface bond strength and the internal force of the supporting member. With the increase in the water–cement ratio, the slurry of broken surrounding rock is filled tightly, and the supporting members are in complete contact with the surrounding rock. However, if the water–cement ratio exceeds the critical value, the cement slurry is thinner and the bonding effect is weakened, so the strength of the supporting member cannot be fully utilized. Therefore, if the water–cement ratio is too large or too small, the interfacial bonding strength between the supporting member and the surrounding rock will be weakened, resulting in the slippage of the supporting member, making it difficult to give full play to its strength potential, and in turn affecting the stability of the whole anchoring–grouting system.

**Figure 7:**
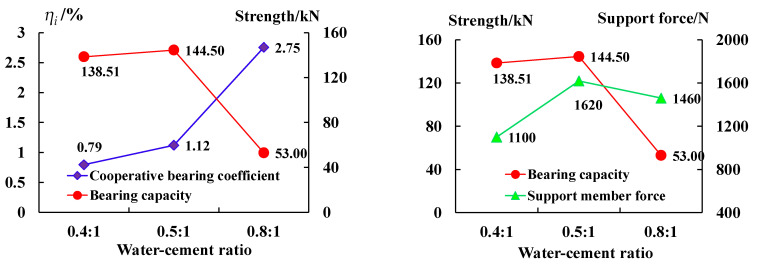
The cooperative bearing curve of the anchoring–grouting system with different water–cement ratios.

Figure 7 shows the relationships between the cooperative bearing coefficient *η_i_*, the bearing capacity of the anchoring–grouting system, and the internal force of the support member. The analysis results show that:

(1) The cooperative bearing coefficient increases with the increase in the water–cement ratio. When the water–cement ratio is 0.8, *ζ_j_* reaches the largest value of 2.75%. When the water–cement ratio is 0.4 and 0.5, *ζ_j_* is 0.79% and 1.12%, respectively.

(2) The bearing capacity relationship of the anchoring–grouting system is the same as that of the supporting members with the change in the water–cement ratio. The bearing capacity of the anchoring–grouting system and the cooperative bearing coefficient is roughly the opposite relationship.

The main reasons are that when the water–cement ratio is appropriate, the slurry can fill the pores, the reinforcement effect of the surrounding rock is significant, the bearing capacity of the anchoring–grouting system is enhanced, the bonding strength between the support component and the anchoring–grouting system interface is increased, and the anchorage of the support component is tight. Therefore, the bearing capacity of the support component is consistent with that of the anchoring–grouting system.

#### 3.1.3. Displacement Variation Relationship of the Support Surface

**Figure 8:**
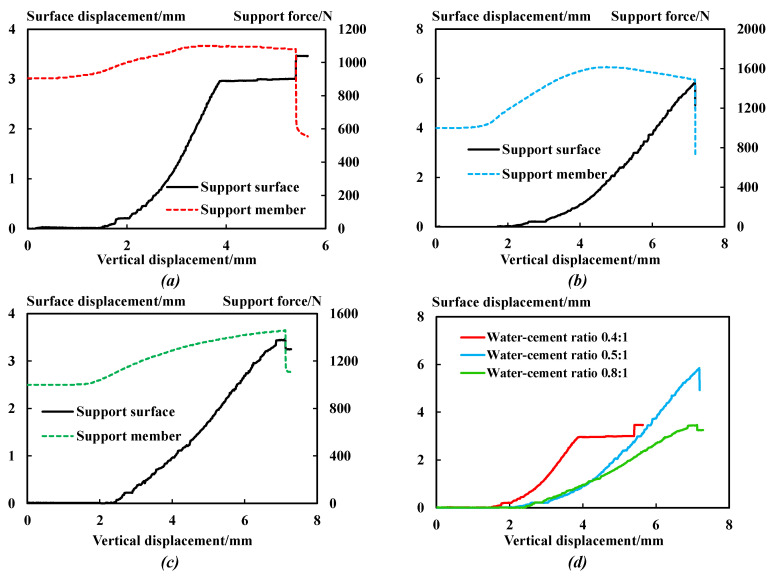
Stress and displacement curve of support. (**a**) Water–cement ratio: 0.4. (**b**) Water–cement ratio: 0.5. (**c**) Water–cement ratio: 0.8. (**d**) Stress comparison curve of supporting components.

Figure 8 shows the horizontal displacement of the anchoring–grouting system support surface and the stress curve of the supporting members.

The comparative analysis shows that:

(1) At the initial loading stage, the stress of the supporting members remains unchanged, and the horizontal displacement of the support surface remains 0. The integrity of the anchoring–grouting system is good with no internal cracks developed.

(2) When the stress of the supporting member reaches the peak value, with the increase in the water–cement ratio, the peak displacement of the anchoring–grouting system support surface first decreases and then increases, which is the opposite of the variation relationship of the peak value of the supporting member.

(3) The supporting members’ stress and the support surface’s horizontal displacement are non-synchronous. With the increase in the stress on the supporting members, the horizontal displacement of the supporting surface stably increases until the supporting members slip and fail. The anchoring–grouting system interacts with the supporting members, increasing the stress on the supporting members and limiting the strength reduction and dilatancy deformation of the anchoring–grouting system [19,20].

(4) When the anchoring–grouting system is loaded to a particular stage, the stress of the supporting members will suddenly drop, the supporting members will slip and fail, and the horizontal displacement of the support surface will suddenly increase or drop. The main reasons are that when the supporting members slip and yield, the support resistance of the supporting members will suddenly decrease, and the constraints on the support surface will reduce, resulting in the sudden increase in the horizontal displacement of the support surface. However, after the sudden drop of support resistance, the binding force on the broken blocks of the support surface decreases, and the fractured blocks fall off, resulting in the sudden drop in horizontal displacement of the support surface.

**Figure 9:**
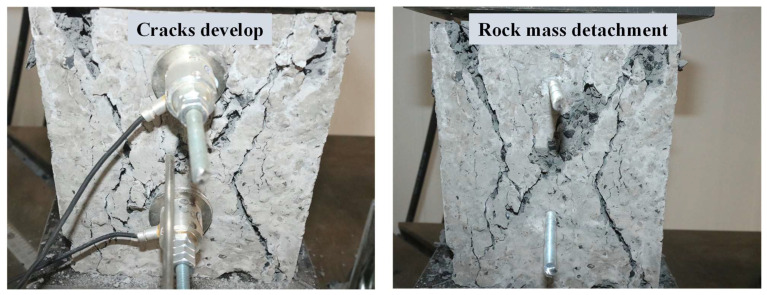
Failure modes of anchor–grouting system specimens.

**Figure 10:**
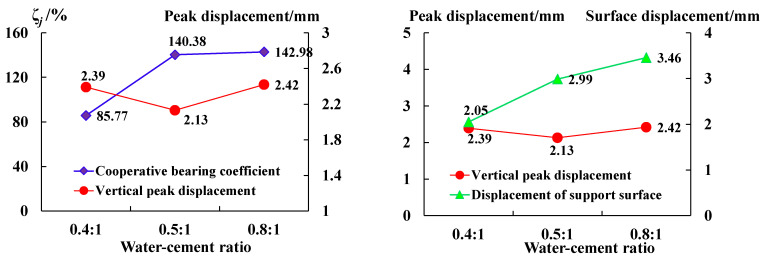
Coordinated deformation coefficient curve of the anchoring–grouting system with different water–cement ratios.

The analysis results in Figure 9 and Figure 10 show that:

(1) The coordinated deformation coefficient of the anchoring–grouting system increases with the water–cement ratio. When the water–cement ratio is 0.8, the coefficient is the largest value of 142.98%. When the water–cement ratio is 0.4 and 0.5, the coefficient is 85.77% and 140.38%, respectively. There is little difference between the coordinated deformation coefficient of the anchoring–grouting system when the water–cement ratio is 0.5 and 0.8.

(2) The displacement of the support surface of the anchoring–grouting system is greater than the peak displacement in the vertical direction of the anchoring–grouting system. The difference between the displacement of the support surface and the peak displacement in the vertical direction increases with the increase in the water–cement ratio. When the water–cement ratio of the slurry is large, the displacement of the roadway support surface is large, and the deformation in the roadway is obvious.

### 3.2. Bearing Mechanical Properties of Anchoring–Grouting System with Different Preloads

#### 3.2.1. Strength Variation Relationship of Anchoring–Grouting System

**Figure 11:**
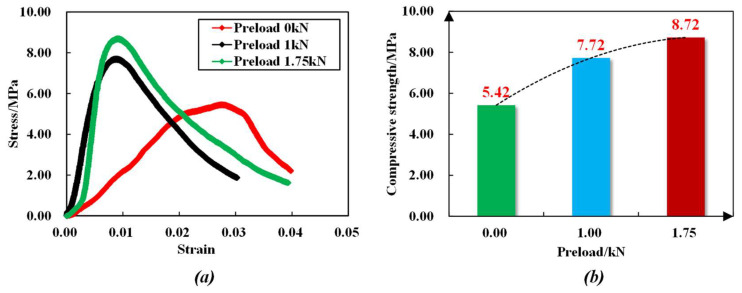
Stress–strain curve of the anchoring–grouting system with different preloads. (**a**) Stress–strain curve. (**b**) Compressive strength curve.

**Figure 12:**
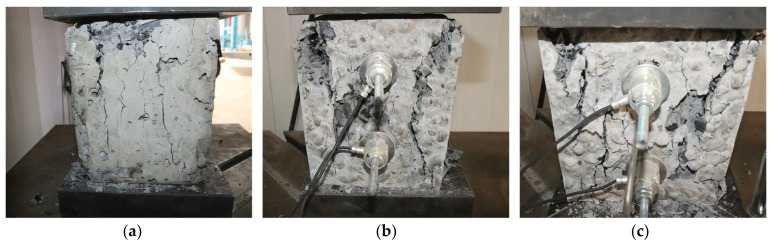
Failure modes of anchoring–grouting system with different preloads. (**a**) Preload: 0 kN. (**b**) Preload: 1 kN. (**c**) Preload: 1.75 kN.

Figure 11 shows the stress–strain relationship curve of the anchoring–grouting system with different preloads, and Figure 12 shows the failure mode of the anchoring–grouting system with different preloads. The research results indicate that:

(1) The compressive strength of the anchoring–grouting system has an apparent increasing trend with the increase in the preload. When the preload increases from 0 kN to 1 kN, the compressive strength of the anchoring–grouting system increases by about 43%. When the preload increases from 0 kN to 1.75 kN, the compressive strength of the anchoring–grouting system increases by about 64%, and there is an obvious linear relationship between the compressive strength of the anchoring–grouting system and the preload.

(2) The supporting member exerts a preload on the anchoring–grouting system, thereby constraining the specimens. The cracks are compacted and closed, which improves the elastic modulus and the bearing capacity, giving full play to the support strength, and realizing the active reinforcement of the anchoring–grouting system. The main crack of the anchoring–grouting system with a preload of 0 kN presents a splitting failure from top to bottom, while that with a preload of 1.75 kN presents an X failure pattern, and a small broken specimen is extruded in the free area between the supporting members.

#### 3.2.2. Stress Variation Relationship of Supporting Members

**Figure 13:**
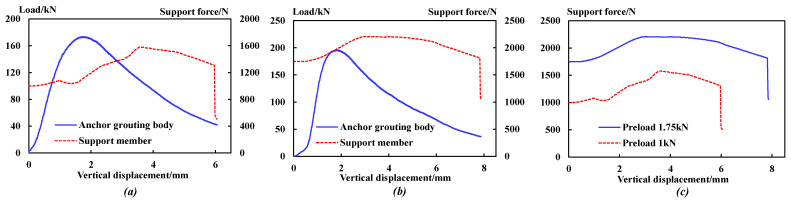
Bearing capacity of anchoring–grouting system and stress curve of supporting component. (**a**) Preload: 1 kN. (**b**) Preload: 1.75 kN. (**c**) Comparison curve.

When the preload is 0 kN, no supporting member is set in the system, so it is impossible to monitor the stress of the supporting member. The stress changes of the supporting members were compared when the preload was 1 kN and 1.75 KN. The bearing capacity of the anchoring–grouting system and the stress curve of the supporting members are shown in Figure 13.

The comparative analysis shows that:

(1) Under different preloads, the force variation relationship of the supporting members is similar. The force of the supporting members can be divided into five stages: steady, rising, stable, falling, and sudden falling.

(2) When the stress of the supporting member drops suddenly, the vertical displacement of the anchoring–grouting system with a preload of 1.75 kN is 7.9 mm, while that of 1 kN is 6.1 mm. A higher preload can give full play to the support strength potential of the components.

(3) When the preload is increased from 1 kN to 1.75 kN, the peak value of the support member internal force increases from 1580 N to 2210 N, but the increase rate of the support members decreases from 58% to 14%.

**Figure 14:**
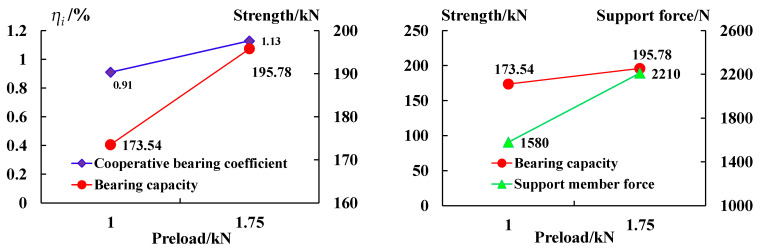
The cooperative bearing curve of the anchoring–grouting system with different preloads.

The analysis in Figure 14 shows that:

(1) The anchoring–grouting system’s cooperative bearing coefficient increases with the initial preload increase. When the initial preload is 1 kN and 1.75 kN, the coefficient is 0.91% and 1.13%, respectively, indicating that the cooperative bearing capacity of the supporting members is enhanced.

(2) With the increase in the initial preload, the bearing capacity of the anchoring–grouting system shows an increasing trend, because the preload results in higher friction between the surrounding rock and the cement and better integrity.

#### 3.2.3. Displacement Variation Relationship of the Support Surface

**Figure 15:**
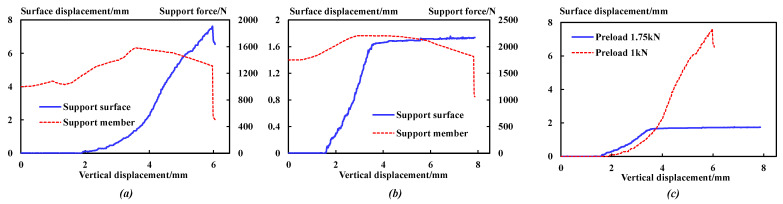
Displacement and force curve of supporting members. (**a**) Preload: 1 kN. (**b**) Preload: 1.75 kN. (**c**) Comparison curve.

Figure 15 is the stress relationship curve between the support surface displacement or support internal force and the vertical displacement under different preloads.

The comparative analysis shows that:

(1) The displacement of the support surface with a preload of 1.0 kN keeps increasing until a sudden drop, when the support force also drops suddenly. However, the displacement of the specimen with a preload of 1.75 kN increases and then remains a constant value, which is much lower than that with a preload of 1.0 kN.

(2) When the preload is increased from 0 kN to 1.75 kN, the peak displacement of the support surface is reduced from 7.56 mm to 1.66 mm. Combined with the force variation relationship of the supporting members, this shows that the preload can effectively inhibit the fragmentation and expansion of the anchoring–grouting system, improve the bearing capacity, and give full play to the support strength.

(3) When the preload of the supporting members is large, the deformation of the roadway support surface can be effectively controlled, and the stability of the surrounding rock can be maintained to ensure the overall safety of deep roadway excavation.

**Figure 16:**
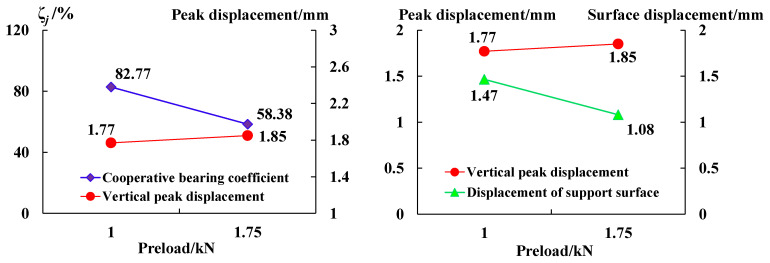
Coordinated deformation coefficient curve of anchoring–grouting system with different preloads.

The comparative analysis in Figure 16 shows that:

(1) The coordinated deformation coefficient of the anchoring–grouting system decreases with the increase in the initial preload. The variation relationship of the vertical peak displacement is the opposite of the coefficient. When the initial preload is 1 kN and 1.75 kN, the coefficient is 82.77% and 58.11%, and the vertical peak displacement is 1.77 mm and 1.85 mm, respectively.

(2) The vertical peak displacement of the anchoring–grouting system is greater than that of the support surface. With the increase in the initial preload, the change in the relationship of the vertical peak displacement of the anchoring–grouting system is the opposite of that of the support surface, indicating that the anchoring preload has a significant effect of surrounding rock deformation control.

## 4. Conclusions

Based on a typical deep excavation coal mine roadway case, we conducted laboratory tests considering water–cement ratios and preloads to study the mechanical characteristics of the anchoring–grouting system in the broken surrounding rock. The conclusions are as follows:

(i) With the increase in the water–cement ratio, the bearing capacity of the anchoring–grouting system first increases and then decreases. With the increase in preload, the bearing capacity has an apparent increasing trend and shows a linear relationship.

(ii) There is a parabolic correlation relationship between the support force and water–cement ratio, and a positive correlation relationship between the support force and preload.

(iii) In the elastoplastic bearing stage of the anchoring–grouting system, the stress of the supporting members begins to increase. In the post-peak failure stage, the stress of the supporting members continues to increase until the peak value and remains stable. The supporting members play an important role after the bearing peak of the anchoring–grouting system.

(iv) The support surface is not deformed in the initial and elastic bearing stages of the anchoring–grouting system. When the stress of the supporting members drops suddenly and fails, the horizontal displacement of the support surface also drops or increases suddenly.

(v) The optimal water–cement ratio and a higher preload can improve the cooperative bearing characteristics of surrounding rock and its support, which is of great significance for enhancing the strength of surrounding rock and reducing roadway deformation.

## Figures and Tables

**Table 1:** Design of test scheme for bearing capacity of anchoring–grouting system.

Scheme No	Water–Cement Ratio	Supporting Member Quantity	Preload/kN
A1	0.4	2	1
A2	0.5	2	1
A3	0.8	2	1
B1	0.5	2	0
B2	0.5	2	1
B3	0.5	2	1.75

## Data Availability

Not applicable.
